# Follistatin Is a Novel Biomarker for Lung Adenocarcinoma in Humans

**DOI:** 10.1371/journal.pone.0111398

**Published:** 2014-10-27

**Authors:** Fangfang Chen, Ping Ren, Ye Feng, Haiyan Liu, Yang Sun, Zhonghui Liu, Jingyan Ge, Xueling Cui

**Affiliations:** 1 Department of Gastrointestinal Surgery, China-Japan Union Hospital, Jilin University, Changchun, China; 2 Department of Thoracic Surgery, First Hospital of Jilin University, Changchun, China; 3 Department of Immunology, College of Basic Medical Sciences, Jilin University, Changchun, China; Cincinnati Children’s Hospital Medical Center, United States of America

## Abstract

**Background:**

Follistatin (FST), a single chain glycoprotein, is originally isolated from follicular fluid of ovary. Previous studies have revealed that serum FST served as a biomarker for pregnancy and ovarian mucinous tumor. However, whether FST can serve as a biomarker for diagnosis in lung adenocarcinoma of humans remains unclear.

**Methods and Results:**

The study population consisted of 80 patients with lung adenocarcinoma, 40 patients with ovarian adenocarcinoma and 80 healthy subjects. Serum FST levels in patients and healthy subjects were measured using ELISA. The results showed that the positive ratio of serum FST levels was 51.3% (41/80), which was comparable to the sensitivity of FST in 40 patients with ovarian adenocarcinoma (60%, 24/40) using the 95th confidence interval for the healthy subject group as the cut-off value. FST expressions in lung adenocarcinoma were examined by immunohistochemical staining, we found that lung adenocarcinoma could produce FST and there was positive correlation between the level of FST expression and the differential degree of lung adenocarcinoma. Furthermore, the results showed that primary cultured lung adenocarcinoma cells could secrete FST, while cells derived from non-tumor lung tissues almost did not produce FST. In addition, the results of CCK8 assay and flow cytometry showed that using anti-FST monoclonal antibody to neutralize endogenous FST significantly augmented activin A-induced lung adenocarcinoma cells apoptosis.

**Conclusions:**

These data indicate that lung adenocarcinoma cells can secret FST into serum, which may be beneficial to the survival of adenocarcinoma cells by neutralizing activin A action. Thus, FST can serve as a promising biomarker for diagnosis of lung adenocarcinoma and a useful biotherapy target for lung adenocarcinoma.

## Introduction

Follistatin (FST), a single chain glycoprotein, is originally isolated from follicular fluid of ovary, which has the effect on inhibiting the secretion of follicle-stimulating hormone (FSH) of pituitary cells [1]. As an activin binding protein, two FST molecules encircle one activin molecule and neutralize activin action by burying its receptor binding sites [2]. Activin, a member of transforming growth factor beta (TGF-β) superfamily, is involved in the acute-phase response, the tissue fibrosis and tumor cell apoptosis [3]–[5]. Previous studies reported that FST participates in various physiological and pathological processes, such as early embryo development [6], establishment of pregnancy [7], ovarian granulosa cell differentiation [8], polycystic ovarian syndrome [9], erythrocyte maturation [10], formation of liver fibrosis and cancer [11]–[12], branching tubules genesis and tubular regeneration after ischemia or reperfusion injury by blocking the action of endogenous activin [13]. Previous studies also revealed that FST protein can be detected not only in the gonads and extragonadal tissues, but also in peripheral blood and cell culture supernatant, and serum FST levels were correlated to pregnancy and cervical cancer [14]–[16].

The most commonly diagnosed cancers worldwide are lung cancer (1.61 million, 12.7% of the total), breast cancer (1.38 million, 10.9%) and colorectal cancer (1.23 million, 9.7%), and the most common cause of cancer death is lung cancer (1.38 million, 18.2% of the total) [17]. Adenocarcinoma is the most common histologic type of lung cancer in most countries, accounting for almost half of all lung cancers [18]. Due to lack of early diagnosis methods, 80% patients with lung adenocarcinoma were found in late stage and have lost the chance of surgical treatment. Thus, to find a novel serum cancer marker has the great significance for early diagnosis of lung adenocarcinoma.

It was reported that lung tumors can produce neuroendocrine hormone, for example, some small cell lung carcinomas secrete adrenocorticotropic hormone (ACTH) [19]–[20]. Our previous studies discovered that ovarian adenocarcinoma can secrete FST [21], but whether lung adenocarcinoma can secrete FST and FST function is still unclear. In search of lung cancer markers, we found that serum FST levels were elevated in some patients of lung adenocarcinoma. In order to clarify the relationship between FST and lung adenocarcinoma, we examined FST levels in serum of patients with lung adenocarcinoma by enzyme-linked immunosorbent assay (ELISA), and analyzed the expression of FST in lung adenocarcinoma tissues by immunohistochemical staining, as well as further investigated the roles of FST in proliferation and apoptosis of lung adenocarcinoma cells.

## Materials and Methods

### Ethics statement

The study was approved by The Ethics Committee of China-Japan Union Hospital of Jilin University, China, and written informed consents were obtained from all participants prior to study entry.

### Patients and Clinical Features

Patients were diagnosed according to the new lung cancer staging system [22]. Serum/cancer tissues of patients with tuberculosis and patients with stage I–III lung adenocarcinoma or stage I–III ovarian mucinous adenocarcinoma were collected sequentially from tissue bank, China-Japan Union Hospital of Jilin University, Changchun, China, between Jan 2009 and Dec 2013. In addition, age-matched healthy subjects attending the routine examinations in the medical examination center of China-Japan Union Hospital of Jilin University were recruited as controls.

### Enzyme-linked immunosorbent assay for FST

Serum samples were obtained according to the previously described methods [21]. All serum samples were collected from patients in the morning of the second day after admission, 2 milliliter of peripheral blood sample was collected from each study subject and serum was obtained by centrifuging at 1500 g for 10 min at 4°C and stored at −80°C until analysed. Serum samples were similarly collected from the healthy subjects in the morning on the day of their routine examination.

Serum FST levels were measured in patients with lung adenocarcinoma, ovarian mucinous adenocarcinoma, lung benign disease tuberculosis and healthy subjects, respectively in triplicate, using ELISA kit (R&D Systems, Minneapolis, USA) according to manufacturer’s instructions. Absorbance was then detected at 450 nm to quantify FST levels.

### Immunohistochemical staining for FST

Ten slides of each type lung adenocarcinoma in well differetiation, moderate differetiation and poor differetiation were obtained from the Department of Pathology at China-Japan Union Hospital of Jilin University for immunohistochemical staining. Immunohistochemical staining for FST was carried out as previously described [21].

### Primary lung adenocarcinoma cell culture

Lung adenocarcinoma tissues and non-tumor lung tissues as control were obtained from patients who underwent a surgical resection. Single cell suspension was prepared by enzyme dispersion [23]. 5×10^5^ cells were plated into 12-well cell culture plates and cultured in Iscove’s Modified Dulbecco’s Medium (IMDM) (GIBCO, CA, USA) supplemented with 10% heat-inactivated fetal calf serum (FCS) (Hyclone, Logan, UT). Cells were incubated at 37°C in a humidified, 95% air and 5% CO_2_ atmosphere for 24 h. Then the cells were washed twice with IMDM, and cultured in IMDM with 2.5% and 10% FCS for 24, 48 and 72 h, respectively. Finally, the supernatant of cultured cells was harvested by centrifuging at 1,500 g for 10 min at 4°C, and stored at −80°C for the measurement of FST levels.

### Human lung adenocarcinoma cells lines culture

Human lung adenocarcinoma cell line A549 was obtained from the American Type Culture Collection (ATCC, USA), and cultured in modified polystyrene T-75 tissue culture flasks in RPMI 1640 (GIBCO, CA, USA) supplemented with 10% FCS at 37°C in a humidified 5% CO_2_ incubator [24].

### Cells proliferation assessed by cell counting kit-8 (CCK8) assay

A549 cells were plated in triplicate in a 96-well plates at 2×10^4^ cells per well and incubated in the absence or presence of various concentrations of recombinant human activin A (R&D, Minneapolis, USA) or anti-FST antibody (1 µg/ml) in 2.5% FCS-RPMI 1640 for 24 h. Cell proliferation was determined by using the cell counting kit-8 (CCK8, Dojindo, Japan) and measured by microplate reader scanning at 450 nm according to the manufacturer’s instruction [25].

### Assay of cells apoptosis by Annexin V-PI analysis

Annexin V-PI staining was used to evaluate A549 cells apoptosis according to the manufacturer’s instructions (Roche, Germany). Briefly, A549 cells were plated in a 12-well cell culture plates at 2×10^5^ cells per well and pre-cultured in 2.5% FCS-RPMI 1640 with or without 1 µg/ml anti-FST monoclonal antibody for 2 h, and then were incubated in the presence of 10 ng/ml activin A for 24 h. A549 cells were stained by Annexin V-Alexa Fluor-488/PI and the stained cells were analyzed by flow cytometry to determine the percentages of AnnexinV+/PI− (early apoptosis) and AnnexinV+/PI+ (late apoptosis) cells. The experiment was repeated 3 times.

### Statistical analysis

Sensitivity for lung cancer was considered as 100×[the number of lung cancer patients with positive tumor marker (true positive)/total number of lung cancer patients (true positive + false negative)] %, specificity for lung cancer as 100×[the number of control healthy subjects with negative tumor marker (true negative)/total number of control healthy subjects (true negative + false positive)] %, [26]. Data were expressed as means ± SD. Results from two groups were compared using two-tailed Student’s t-test. P-value≤0.05 was considered to be statistically significant.

## Results

### Clinical Features of Patients

The main study participants comprised 80 healthy subjects, 40 patients with stage I–III ovarian mucinous adenocarcinoma and 80 patients with stage I–III lung adenocarcinoma. Demographic and clinical characteristics of the study participants are given in Table 1. There was no statistical difference (p>0.05) in age between any of the groups studied, and the smoker number, the average number of cigarettes smoked per day and the duration of smoking was no difference between lung adenocarcinoma cases and controls (p>0.05).

**Table 1:** Demographic characteristics and clinical data for healthy subjects, patients with lung and ovarian mucinous adenocarcinoma.

		Healthy subjects	Lung adenocarcinoma	Ovarian adenocarcinoma
Age (years)				
	Mean ± SD	53.7±10.9	57.8±6.5	55.9±7.4
	Range	35–65	31–73	39–72
Sex (n)				
	female	40	34	40
	male	40	46	-
Pathologic Stage (*n*)				
	I	-	14	5
	II	-	30	18
	III	-	36	17
Differentiation (n)				
	Well	-	11	9
	Moderately	-	32	15
	poorly	-	37	16
Total (n)		80	80	40

Note: No statistical differences in age were found between any of the groups studied (P>0.05). Risk factors such as smoking are not considered to influence our research data because the numbers of subjects from different groups are similar.

### Serum FST levels

**Figure 1:**
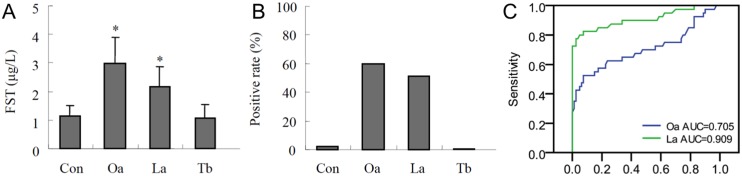
The levels of serum FST in health subjects and patients. (A) The serum FST levels were examined by ELISA. (B) The positive ratio of serum FST levels were represented in health subjects and patients using the 95th confidence interval in the healthy subjects as the cut-off value. Cont, health subjects control; Oa, ovarian mucinous adenocarcinoma; La, lung adenocarcinoma; Tb, tuberculosis. *P<0.01, compared with health control group. (C) Receiver-operating characteristic (ROC) curve for FST for the diagnosis of lung adenocarcinoma and ovarian cancer. AUC: the area under the curve.

By assaying serum FST levels, we found that it was significantly higher in patients with lung adenocarcinoma and ovarian mucinous adenocarcinoma than that in healthy control group (P<0.01, Figure 1A). In contrast, the serum FST levels had no significant difference between patients with lung benign disease tuberculosis and healthy control group (P>0.1). Using the 95th confidence interval in the healthy subjects as the cut-off value, the sensitivity of serum FST levels was 51.3% (41/80) in patients with lung adenocarcinoma and 60% (24/40) in patients with ovarian mucinous cancer (Figure 1B). When the sensitivity and specificity of FST in patients with lung adenocarcinoma and ovarian mucinous cancer were plotted on a ROC curve (Figure 1C), the area under the curve was 0.705 and 0.909 respectively. These data indicated that serum FST levels might be a useful biomarker for diagnosis of lung adenocarcinoma.

### Relationship between serum FST levels and stage, grade of lung adenocarcinoma

Using the 95th confidence interval in the healthy subjects as the cut-off value, there is no difference in the positive ratio of serum FST levels between female (50%) and male (52.2%) patients with lung adenocarcinoma. In contrast, the positive ratio of serum FST levels were related to disease stages and differentiation grades of lung adenocarcinoma and ovarian mucinous adenocarcinoma (table 2).

**Table 2:** The positive ratio of serum FST levels in healthy subjects, patients with lung and ovarian mucinous adenocarcinoma.

		Healthy subjects	Lung adenocarcinoma	Ovarian adenocarcinoma
Sex				
	female	5% (2 in 40)	50% (17 in 34)	60% (24 in 40)
	male	0 (0 in 40)	52.1% (24 in 46)	
Pathologic Stage (n)				
	I	-	42% (6 in 14)	40% (2 in 3)
	II	-	53% (16 in 30)	61.1% (11 in 18)
	III	-	53% (19 in 36)	64, 7% (11 in 17)
Differentiation (n)				
	Well	-	36% (4 in 11)	55.6% (5 in 9)
	Moderate	-	38% (12 in 32)	60% (9 in 15)
	poor	-	68% (25 in 37)	62.5% (10 in 16)
Positive ratio (n)		2.5% (2 in 80)	51.3% (41 in 80)	60% (24 in 40)

### Expression of FST in lung adenocarcinoma tissues

**Figure 2:**
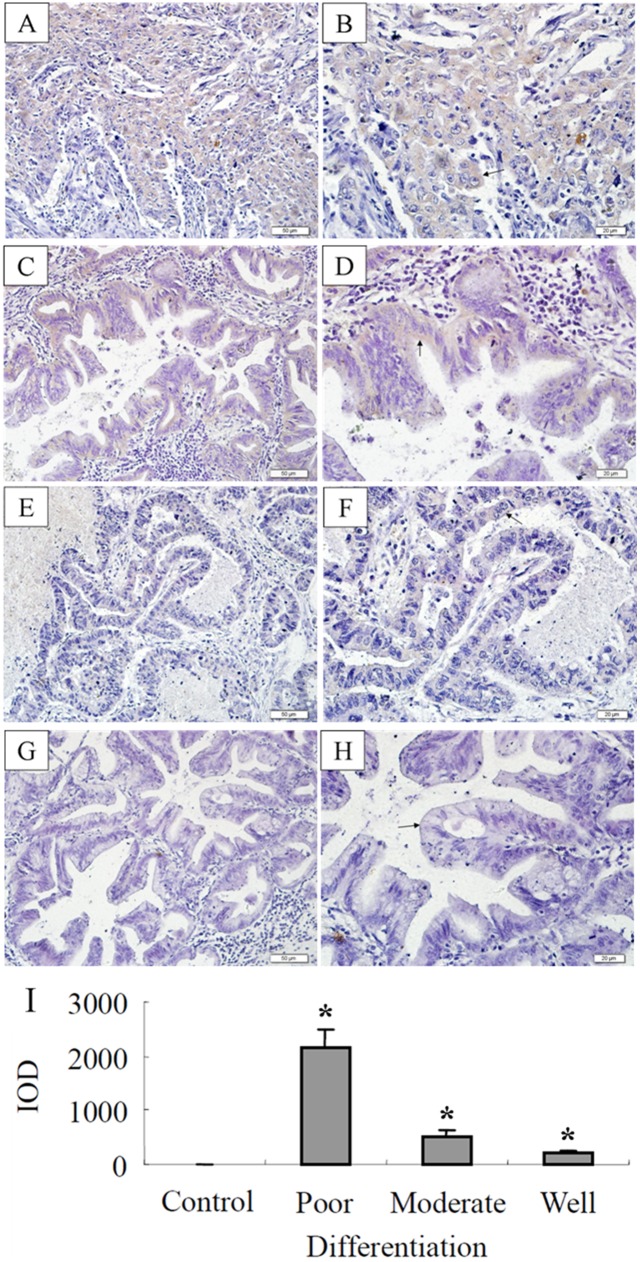
FST expression in lung adenocarcinoma tissues. (A, B) The poorly differentiated, (C, D) moderately differentiated and (E, F) well differentiated lung adenocarcinoma were stained by anti-FST monoclonal antibody, respectively. The arrows in A–F represented the positive staining area for FST. (G, H) No positive staining area was found with normal mouse IgG instead of anti-FST monoclonal antibody as a procedural background control. The graph represents the integrated optical density (IOD) value obtained using IMAGEJ (http://rsbweb.nih.gov/ij/).

To confirm FST expression in lung adenocarcinoma, immunohistochemical staining was performed to examine FST protein. The results showed FST immunoreactivity in the glandular duct-like tissues of lung adenocarcinoma. The expression of FST in lung adenocarcinoma was correlated with the differentiation, that is to say, its expression became more pronounced as the grade became worse, i.e. from well-differentiated to poorly-differentiated lung adenocarcinoma (Fig. 2). Using ELISA to detect serum FST, we also found that the positive ratio of serum FST levels in patients with lung adenocarcinoma was correlated with the differentiation grade of lung adenocarcinoma (Table 2), which was similar to the results of immunohistochemical staining.

### FST production from lung adenocarcinoma cells

**Figure 3:**
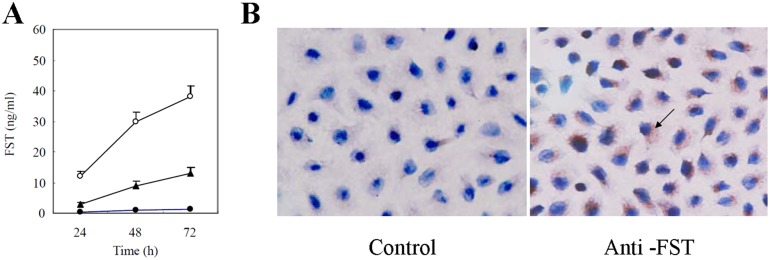
The production of FST from lung adenocarcinoma cells. (A) The tumor cells were incubated in 2.5% and 10% FCS-IMEM culture medium, respectively. The non-tumor lung tissue cells were incubated in 10% FCS-IMEM culture medium. The supernatants of cultured cells were harvested at the indicated time point and FST levels were detected by ELISA. ○ Lung adenocarcinoma cells with 10% FCS-IMDM medium. Δ Lung adenocarcinoma cells with 2.5% FCS-IMDM medium. • Non-tumor lung tissue cells with 10% FCS-IMEM medium. (B) FST protein expression in human lung adenocarcinoma cell line A549 cells was examined by immunocytochemical staining with anti-FST antibody (Anti-FST). A procedural background control (Control) was performed using normal mouse IgG instead of anti-FST antibody.

To clarify FST production in lung adenocarcinoma, cells obtained from lung adenocarcinoma tissues and non-tumor lung tissues were cultured *in vitro* and FST levels in the supernatants of cultured cells were detected using ELISA. As shown in Figure 3A, FST production was identified in the supernatant of cultured lung adenocarcinoma cells, but was almost not found in the supernatant of control cells derived from non-tumor lung tissues in 10% FCS-IMDM. Furthermore, the immunocytochemical staining revealed that human lung adenocarcinoma cell line A549 also produced FST protein (Fig. 3B). These data suggested that human lung adenocarcinoma cells secrete FST and the increased FST in serum of patients with lung adenocarcinoma might be produced by lung adenocarcinoma cells.

### Effects of FST on A549 proliferation

**Figure 4:**
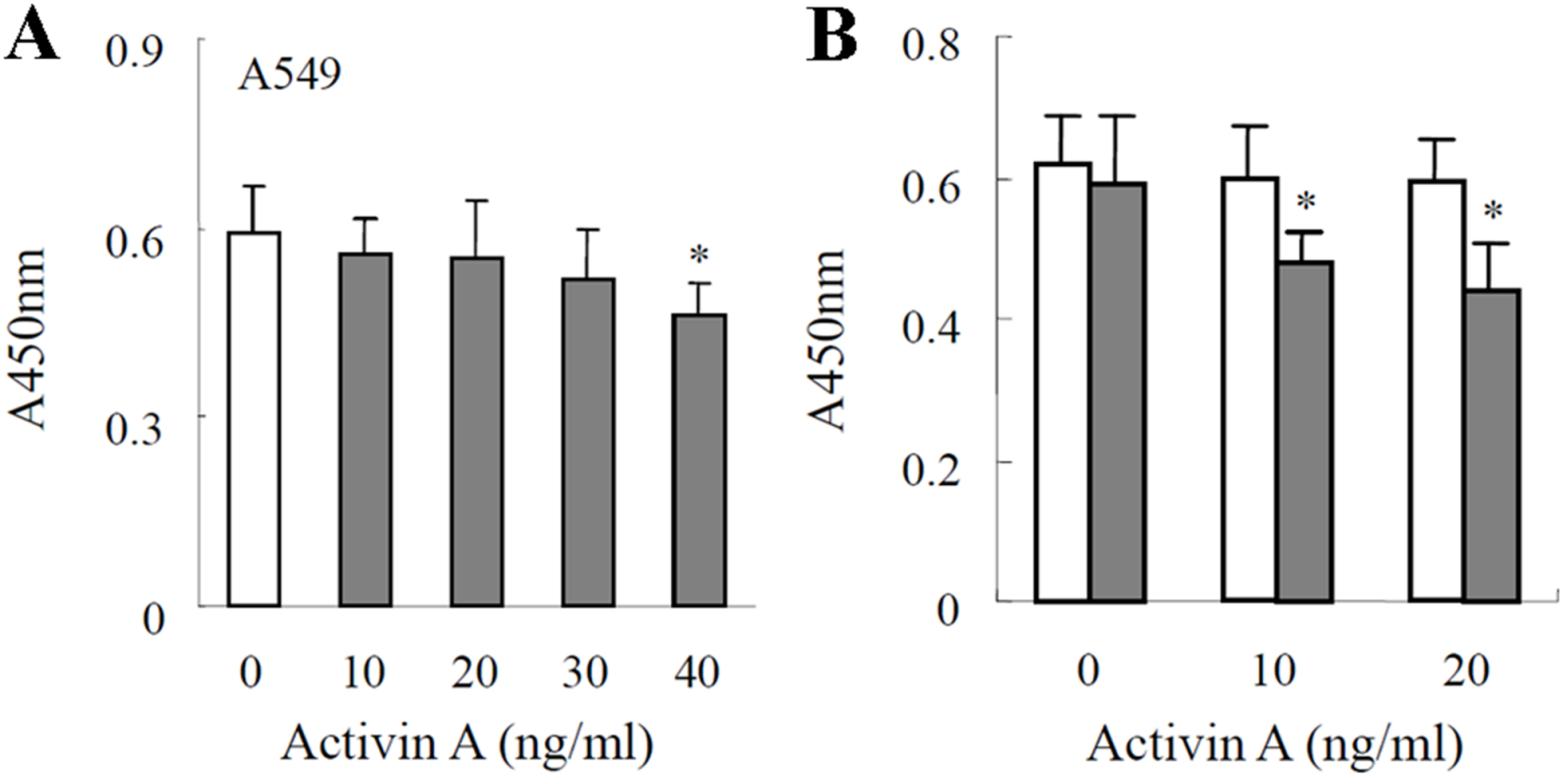
The effect of anti-FST antibody on proliferation of lung adenocarcinoma cells. (A) Proliferation of lung adenocarcinoma A549 cells treated with 0–40 ng/ml activin A for 24 h was determined by using the cell counting kit-8. Absorbance was detected at 450 nm to express the cell viabilities. *P<0.05 compared with 0 controls. (B) A549 cells were incubated in the presence of 0–20 ng/ml activin A (□) or 1 µg/ml anti-FST monoclonal antibody + 0–20 ng/ml activin A (▪).*P<0.05, compared with activin A group and 0 control.

Previous studies have reported that activin A can inhibit proliferation and induce apoptosis of lung cancer cells [5]. As an activin binding protein, FST can neutralize activin A action by burying its receptor binding sites [27]. Therefore, the proliferation of A549 cells was assessed by CCK8 assay. The results showed that after cultured A549 cells with different concentrations activin A (0–40 ng/ml) for 24 h, 40 ng/ml activin A could significantly inhibit the proliferation of A549 cells compared with untreated cells (P<0.05, Fig. 4A). However, when anti-FST monoclonal antibody was applied to neutralize endogenous FST, even 10 ng/ml activin A could also remarkably inhibit the proliferation of A549 cells (Fig. 4B). These results indicated that endogenous FST produced by lung adenocarcinoma cells might promote survival of tumor cells by neutralizing activin bioactivities.

### Role of FST in A549 cells apoptosis

**Figure 5:**
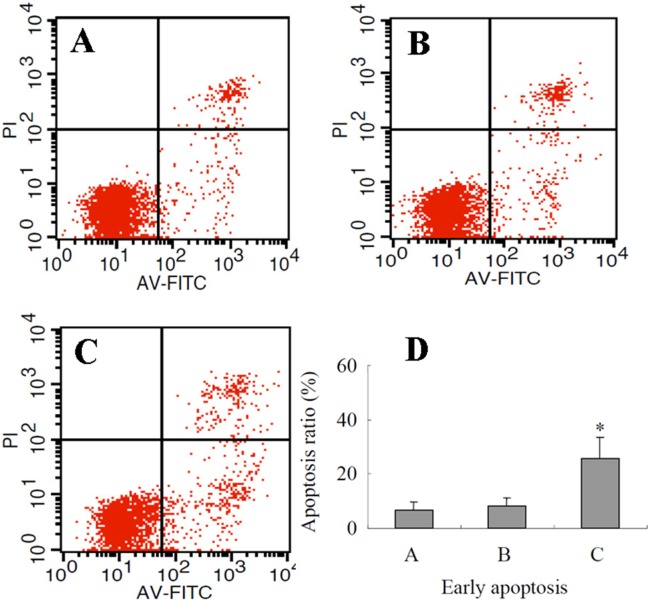
The effect of anti-FST antibody on apoptosis of activin A-induced lung adenocarcinoma cell line A549 cells. A549 cells were incubated for 24 hours in the absence of activin A (A), presence of 10 ng/ml activin A (B) or 1 µg/ml anti-FST monoclonal antibody + 10 ng/ml activin A (C). A549 cells were stained by Annexin V-Alexa Fluor-488/PI and the stained cells were analyzed by flow cytometry to determine the percentages of AnnexinV^+^/PI^−^ (early apoptosis) and AnnexinV^+^/PI^+^ (late apoptosis) cells. The graph represents means and S.D of triplicate determinations. *P<0.01, compared with 10 ng/ml activin A group and 0 control.

To further investigate the biological role of FST, we analyzed the effect of FST on activin A-induced lung adenocarcinoma cell apoptosis. The results showed that after lung adenocarcinoma cells were cultured only in the presence of 10 ng/ml activin A for 24 h, there was no significant apoptosis of lung adenocarcinoma cells compared with control (P>0.05, Fig. 5). While after using anti-FST monoclonal antibody to neutralize endogenous FST, 10 ng/ml activin A could significantly induced apoptosis of lung adenocarcinoma cells (*P*<0.01).

## Discussion

Based on the global cancer statistics, lung cancer was the most commonly diagnosed cancer and the leading cause of cancer death in males in 2008 globally, and among females, it was the fourth most commonly diagnosed cancer and the second leading cause of cancer death [17]. The poor prognosis is attributable to lack of efficient early diagnostic methods and successful treatment for lung cancer. In the Currently, diagnostic methods for lung cancer mainly based on X-ray imaging, percutaneous biopsy, bronchoscope and sputum cytology, etc. Some studies have reported that levels of serum carcinoembryonic antigen (CEA), cytokeratin 19 fragment (CYFRA21-1) and other tumor markers are elevated in some patients with lung cancer, but the sensitivity was about 30%–50% and the specificity is about 60–80% [26], [28]–[29]. In additional, most squamous cell carcinomas are located at the central bronchus of lung, which can be easier diagnosed by bronchoscopical biopsy and/or brush slices and/or sputum cytology. But most common adenocarcinomas in lung cancer are located at the peripheral lung tissues, which only could be diagnosed by percutaneous pleural biopsy puncture. 80% patients with lung adenocarcinoma are in later stage when the cancers are diagnosed and the patients lose the chance of surgical treatment. Serological tests are convenient and easy, but there is still no specific and sensitive serum marker for lung cancer diagnosis.

Planque and his coleagues [30] reported that FST elevated in lung cancer, but they did not mention the information of FST expression in different histological backgrounds. We examined serum FST in patients with lung cancers in different histological backgrounds, including adenocarcinoma, squamous carcinoma, small cell lung carcinoma. We found that serum FST levels were highest in patients with adnocarcinoma and had no diagnostic value in squamous carcinoma and small cell lung carcinoma (data not shown). In order to further clarify the clinical significance of FST, the expression of FST protein in tissues of lung adenocarcinoma with different grades was analyzed by immunohistochemical staining. The results showed that the expression of FST in lung adenocarcinoma was correlated with the differentiation of lung adenocarcinoma. The lowest FST expression was observed in well differentiated lung adenocarcinoma, and the highest FST expression in poorly differentiated lung adenocarcinoma. The data above suggested that FST is a useful biomarker for the diagnosis of lung adenocarcinoma and a sensitive indicator for the differential grade of lung adenocarcinoma, but FST cannot be a diagnostic biomarker for lung squmous cancinoma and small cell lung cancer.

To confirm FST production from lung adenocarcinoma, cells derived from lung adenocarcinoma and non-tumor lung tissue were cultured and the levels of FST in the supernatant were detected by ELISA. The data revealed that lung adenocarcinoma cells secreted FST in FCS-dependent and time-dependent manner, while cells from non-tumor lung tissues could not secrete FST. Lung adenocarcinoma cells secreted FST in a serum-dependent manner, which is consistent with the FST secretion of pituitary cells as described previously [14]. These findings suggested that elevated FST in serum of patients with lung adenocarcinoma may be directly secreted by adenocarcinoma cells.

Activin has very extensive bioactivities, such as cell differentiation, proliferation, apoptosis and regulation of immune responses [3], [5], [31]–[34]. As an activin binding protein, FST can inhibit the function of activin A [2], and imbalance of activin A and FST expressions is one of the important factors for tumor genesis. Previous studies showed that activin A can induce lung adenocarcinoma cell line A549 cells apoptosis, and FST can weaken the activin A-induced A549 cell apoptosis [5]. In the present study, we found that 10 ng/ml activin A could not inhibit the proliferation of lung adenocarcinoma A549 cells and induce apoptosis of lung adenocarcinoma A549 cells. But neutralizing endogenous FST with anti-FST antibody could increase the effect of activin A on inhibiting A549 cells proliferation. Furthermore, flow cytometry assays showed that neutralizing endogenous FST with anti-FST antibody significantly promoted activin A action on inducing apoptosis of A549 cells. The results indicated that FST might promote the growth of lung adenocarcinoma by inhibiting activin A-inducing apoptosis of adenocarcinoma cells. Hirokazu Oligo et al reported that FST suppresses small cell lung cancer multiple-organ metastasis by inhibiting the angiogenesis [35]. The conclusion of this article does not conflict with ours, small cell lung cancer is different from no-small cell lung cancer, such as small cell lung is only sensitive to radiotherapy, but no-small cell lung cancer is sensitive to both of chemotherapy and radiotherapy. Some articles show that FST may facilitate prostate cancer cell proliferation invasion and metastasis [36]. Additionally, Bing Wang et al reports that FST inhibits activin A inducing A549 cell apoptosis [5], their conclusion supports the effect of FST in lung adenocarcinoma that we found and is different from the effect of FST in small cell lung cancer that Hirokazu Oligo found. This is because lung adenocarcinoma and small cell lung cancer have different pathological type.

In conclusion, the data in our present studies demonstrated that the elevated FST in serum of patients with lung adenocarcinoma was directly produced by lung adenocarcinoma cells, and might promote the survival of lung adenocarcinoma cells. Thus FST may be an important biomarker for diagnosis of lung adenocarcinoma in humans and a useful target for lung adenocarcinoma biotherapy in humans.

## References

[1] Robertson DM, Klein R, de Vos FL, Mclachlan RI, Wettenhall RE, et al (1987) The isolation of polypeptides with FSTH suppressing activity from bovine follicular fluid which are structurally different to inhibin. Biochem Biophys Res Commun 149: 744–749. 3122741 10.1016/0006-291x(87)90430-x

[2] Thompson TB, Lerch TF, Cook RW, Woodruff TK, Jardetzky TS (2005) The structure of the follistatin: activin complex reveals antagonism of both type I and type II receptor binding. Dev Cell 9: 535–543. 16198295 10.1016/j.devcel.2005.09.008

[3] Kim YI, Kim BH, Khang I, Cho BN, Lee HK (2009) Cell growth regulation through apoptosis by activin in human gastric cancer SNU-16 cell lines. Oncol Rep 21: 491–497. 19148527

[4] Wang Y, Cui X, Tai G, Ge J, Li N, et al (2009) A critical role of activin A in maturation of mouse peritoneal macrophages in vitro and in vivo. Cell Mol Immunol 6: 387–392. 19887052 10.1038/cmi.2009.50 PMC4003222

[5] Wang B, Feng Y, Song X, Liu Q, Ning Y, et al (2009) Involvement of ERK, Bcl-2 family and caspase 3 in recombinant human activin A-induced apoptosis in A549. Toxicology 258: 176–183. 19428937 10.1016/j.tox.2009.01.023

[6] VandeVoort CA, Mtango NR, Lee YS, Smith GW, Latham KE (2009) Differential effects of follistatin on nonhuman primate oocyte maturation and pre-implantation embryo development in vitro. Biol Reprod 81: 1139–1146. 19641179 10.1095/biolreprod.109.077198 PMC2802231

[7] Florio P, Gabbanini M, Borges LE, Bonaccorsi L, Pinzauti S, et al (2010) Activins and related proteins in the establishment of pregnancy. Repro Sci 17: 320–330. 10.1177/1933719109353205 20228378

[8] Nakamura T, Hasegawa Y, Sugino K, Koqawa K, Titani K, et al (1992) Follistatin inhibits actvin-induced diffrenciation of rat follistatin granulosa cells in vitro. Biochem Biophys Acta 1135: 103–109. 1591267 10.1016/0167-4889(92)90173-9

[9] Lambert-Messerlian G, Taylor A, Leykin L, Isaacson K, Toth T, et al (1997) Characterization of intrafollicular steroid hormones, inhibin, and follistatin in women with and without polycystic ovarian syndrome following gonadotropin hyperstimulation. Biol Reprod. 57: 1211–1216. 9369189 10.1095/biolreprod57.5.1211

[10] Maguer-Satta V, Bartholin L, Jeanpierre S, Ffrench M, Martel S, et al (2003) Regulation of human erythropoiesis by activin A, BMP2, and BMP4, members of the TGFB family. Exp Cell Res 282: 110–120. 12531697 10.1016/s0014-4827(02)00013-7

[11] Patella S, Phillips DJ, Tchongue J, de Kretser DM, Sievert W (2006) Follistatin attenuates early liver fibrosis: effects on hepatic stellate cell activation and hepatocyte apoptosis. Am J Physiol Gastrointest Liver Physiol. 290: G137–144. 16123203 10.1152/ajpgi.00080.2005

[12] Tumminello FM, Badalamenti G, Fulfaro F, Incorvaia L, Crescimanno M, et al (2010) Serum follistatin in patients with prostate cancer metastatic to the bone. Clin Exp Metastasis. 27: 549–555. 20623366 10.1007/s10585-010-9344-x

[13] Kojima I, Maeshima A, Zhang YQ (2001) Role of the activin-follistatin system in the morphogenesis and regeneration of the renal tubules. Mol Cell Endocrinol. 180: 179–182. 11451589 10.1016/s0303-7207(01)00511-1

[14] Liu ZH, Shintani Y, Sakamoto Y, Harada K, Zhang CY, et al (1996) Effects of LHRH, FSH and activin A on follistatin secretion from cultured rat anterior pituitary cells. Endocr J. 43: 321–327. 8886627 10.1507/endocrj.43.321

[15] Wakatsuki M, Shintani M, Abe M, Liu Z, Shitsukawa K, et al (1996) Immunoradiometric assay for follistatin: serum immunoreactive follistatin level in normal adult and pregnant women. J Clin Endocrinol Metab. 81: 630–634. 8636280 10.1210/jcem.81.2.8636280

[16] Gao X, Wei S, Lai K, Sheng J, Su J, et al (2010) Nucleolar follistatin promotes cancer cell survival under glucose-deprived conditions through inhibiting cellular rRNA synthesis. J Biol Chem 285: 36857–36864. 20843798 10.1074/jbc.M110.144477 PMC2978615

[17] Ferlay J, Shin HR, Bray F, Forman D, Mathers C, et al (2010) Estimates of worldwide burden of cancer in 2008: GLOBOCAN 2008. Int J Cancer 127: 2893–2917. 21351269 10.1002/ijc.25516

[18] Parkin DM, Ferlay J, Curado MP, Bray F, Edwards B, et al (2010) Fifty years of cancer incidence: CI5 I–IX. Int J Cancer. 127: 2918–2927. 21351270 10.1002/ijc.25517

[19] Ejaz S, Vassilopoulou-Sellin R, Busaidy NL, Hu MI, Waquespack SG, et al (2011) Cushing syndrome secondary to ectopic adrenocorticotropic hormone secretion: the University of Texas MD Anderson Cancer Center Experience. Cancer 117: 4381–4389. 21412758 10.1002/cncr.26029 PMC3134535

[20] Pedro R, Jose LC, Margarida D, Davide C (2012) Ectopic cushing’s syndrome caused by a pulmonary ACTH-secreting tumor in a patient treated with octreotide. Arq Bras Endocrinol Metabol 56: 461–464. 23108752 10.1590/s0004-27302012000700009

[21] Ren P, Chen FF, Liu HY, Cui XL, Sun Y, et al (2012) High serum levels of Follistatin in patients with ovarian cancer. J Int Med Res 40: 877–886. 22906260 10.1177/147323001204000306

[22] Detterbeck FC, Boffa DJ, Tanoue LT (2009) The new lung cancer staging system. Chest 136: 260–271. 19584208 10.1378/chest.08-0978

[23] Farrell WE, Clark AJ, Stewart MF, Crosby SR, White A (1992) Bromocriptine inhibits pro-opiomelanocortin mRNA and ACTH precursor secretion in small cell lung cancer cell lines. J Clin Invest 90: 705–710. 1325994 10.1172/JCI115941 PMC329920

[24] Morgillo F, Cascone T, D’Aiuto E, Martinelli E, Troiani T, et al (2011) Antitumour efficacy of MEK inhibitors in human lung cancer cells and their derivatives with acquired resistance to different tyrosine kinase inhibitors. Br J Cancer 105: 382–392. 21750552 10.1038/bjc.2011.244 PMC3172903

[25] Ying X, Cheng S, Wang W, Lin Z, Chen Q, et al (2011) Effect of boron on osteogenic differentiation of human bone marrow stromal cells. Biol Trace Elem Res 144: 306–315. 21625915 10.1007/s12011-011-9094-x

[26] Okamura K, Takayama K, Izumi M, Harada T, Furuyama K, et al (2013) Diagnostic value of CEA and CYFRA 21-1 tumor markers in primary lung cancer. Lung Cancer 80: 45–49. 23352032 10.1016/j.lungcan.2013.01.002

[27] De Winter JP, ten Dijke P, de Vries CJ, van Achterberg TA, Suqini H, et al (1996) Follistatins neutralize activin bioactivity by inhibition of activin binding to its type II receptors. Mol Cell Endocrinol. 116: 105–114. 8822271 10.1016/0303-7207(95)03705-5

[28] Ardizzoni A, Cafferata MA, Tiseo M, Filiberi R, Marroni P, et al (2006) Decline in serum carcinoembryonic antigen and cytokeratin 19 fragment during chemotherapy predicts objective response and survival in patients with advanced non-small cell lung cancer. Cancer 107: 2842–2849. 17103443 10.1002/cncr.22330

[29] Wang WJ, Tao Z, Gu W, Sun LH (2013) Clinical observations on the association between diagnosis of lung cancer and serum tumor markers in combination. Asian Pac J Cancer Prev 14: 4369–4371. 23992005 10.7314/apjcp.2013.14.7.4369

[30] Planque C, Kulasingam V, Smith CR, Reckamp K, Goodglick L, et al (2009) Identification of five candidate lung cancer biomarkers by proteomics analysis of conditioned media of four lungcancer cell lines. Mol Cell Proteomics 8: 2746–2758. 19776420 10.1074/mcp.M900134-MCP200 PMC2816016

[31] Licona-Limon P, Aleman-Muench G, Chimal-Monroy J, Macias-Silva M, Garcia-Zepeda EA, et al (2009) Activins and inhibins: novel regulators of thymocyte development. Biochem Biophys Res Commun 381: 229–235. 19338778 10.1016/j.bbrc.2009.02.029 PMC2693414

[32] Nishihara T, Okahashi N, Ueda N (1993) Activin A induces apoptotic cell death. Biochem Biophys Res Commun 197: 985–991. 8267637 10.1006/bbrc.1993.2576

[33] Aleman-Muench GR, Soldevila G (2012) When versatility matters: activins/inhibins as key regulators of immunity. Immunol Cell Biol 90: 137–148. 21537340 10.1038/icb.2011.32

[34] Li N, Cui X, Ge J, Niu L, Liu H, et al (2013) Activin A inhibits activities of lipopolysaccharide- activated macrophages via TLR4, not of TLR2. Biochem Biophys Res Commun 435: 222–228. 23665022 10.1016/j.bbrc.2013.04.077

[35] Ogino H, Yano S, Kakiuchi S, Muguruma H, Ikuta K, et al (2008) Follistatin suppresses the production of experimental multiple-organ metastasis by small cell lung cancer cells in natural killer cell-depleted SCID mice. Clin Cancer Res 14: 660–667. 18245525 10.1158/1078-0432.CCR-07-1221

[36] Sepporta MV, Tumminello FM, Flandina C, Crescimanno M, Giammanco M, et al (2013) Follistatin as potential therapeutic target in prostate cancer. Targ Oncol 8: 215–223. 10.1007/s11523-013-0268-7 23456439

